# Brain Gray Matter Changes Associated with Mindfulness Meditation in Older Adults: An Exploratory Pilot Study using Voxel-based Morphometry

**DOI:** 10.17140/NOJ-1-106

**Published:** 2014-11-12

**Authors:** Florian Kurth, Eileen Luders, Brian Wu, David S. Black

**Affiliations:** 1 Department of Neurology, UCLA School of Medicine, Los Angeles, CA 90095, USA; 2 USC School of Medicine, Los Angeles, CA 90032, USA; 3 Department of Preventive Medicine, USC School of Medicine, Los Angeles, CA 90032, USA

**Keywords:** Mindfulness, Meditation, Aging, Brain imaging, Voxel-based morphometry

## Abstract

**Background:**

Mindfulness-based interventions (MBIs) have previously been associated with structural gray matter changes in normal healthy adults. However, it remains unknown if standardized MBIs can induce similar changes in older adults and those with health complaints as well. The objective of this investigation was to examine the effect of a standardized MBI on the gray matter tissue of older adults with sleep disturbances.

**Methods:**

This exploratory single-group pilot longitudinal study examined local gray matter changes over a six-week MBI period. Participants included six older adult community volunteers (M=66.5 years of age, SD=5.5, range=58–75; 66% female) with sleep disturbances recruited through advertisement in local newspapers/flyers posted at a university medical center and affiliated clinics in Los Angeles, CA. The MBI was delivered as a weekly, two-hour, six-session, group-based course in mindfulness meditation. Gray matter was measured voxel-wise pre- and post-intervention.

**Results:**

A significant gray matter increase was identified within the precuneus, possibly implicating meditation-induced changes of the default mode network. In contrast, observed significant gray matter decreases may have been driven by MBI-related remediation of brain architecture subserving sleep complaints.

**Conclusions:**

Exploratory findings suggest that mindfulness meditation practice is associated with a detectable alteration of cerebral gray matter in older adults.

## INTRODUCTION

Mindfulness-based Interventions (MBIs) train participants in formalized and systematic mainstreamed mindfulness meditation practices, which are shown to impart significant improvements in stress-related ailments in age groups spanning childhood^1^ to adulthood.^2^ Mind-body practices are more recently showing utility for improving the health of older adults.^3,4^ Neural mechanisms, both functional and structural, may underlie some of these observed benefits. As shown with magnetic resonance imaging, MBIs as brief as eight weeks can modulate the brain structure of young and mid-aged adults,^5,6^ and several cross-sectional studies note significant links between mindfulness practice and gray matter configuration.^7–10^ However, as the effects of MBIs on brain structure have been predominantly investigated in younger and middle-aged cohorts, it remains unknown if and how MBIs impact brain structure specifically in older adults.

In this exploratory, data-generating study, we examine pre-to-post changes in cerebral gray matter volume in older adults with sleep disturbance symptoms who participated in a six-week formalized MBI. We applied voxel-based morphometry to detect potential significant changes in gray matter volume with an extremely high regional specificity (i.e., voxel by voxel) across the entire brain. To our knowledge, this is the first study focused solely on older adults to examine brain changes associated with participation in a mindfulness meditation program.

## METHODS

### Participants and Procedures

Participants included six older adult community volunteers (*M*=66.5 years of age, *SD*=5.5, range=58–75; 66% female; 100% Caucasian) recruited through advertisement in local newspapers/flyers posted at a university medical center and affiliated clinics in Los Angeles, CA, U.S.A. Participants were a subsample from a larger trial that examined the efficacy of a MBI on sleep complaints.^11^ Except for the six subjects described here, the remainder of the sample did not undergo brain scans. Participants were eligible for the study if they were >55 years old, spoke English, and experienced current sleep complaints (Pittsburg Sleep Quality Index >5),^12^ and spoke English. Participants were ineligible if they had a significant current practice of any form of meditation (>15 minutes daily) or depression (Patient Health Questionnaire>14).^13^ The UCLA Institutional Review Board approved all study procedures. Eligible respondents provided written informed consent prior to enrolling in the study. Eight visits to the study site were requested to complete the study protocol, including 1 pre-intervention brain scan, 6 MBI sessions, and 1 post-intervention brain scan. All scans were administered within 10 days pre- and post-intervention. Assessment visits included survey completion and MRI safety screening.

## INTERVENTION

### Mindful Awareness Practices for Daily Living (MAPs)

MAPs is a weekly, 2-hour, 6-session, group-based course in mindfulness meditation (see http://marc.ucla.edu). A certified teacher with over 20 years of mindfulness practice delivered the formalized program curriculum to study participants. An average of 10 to 30 minutes of mindful experiential practice is engaged in each class in addition to the teacher-delivered didactic material and group discussion. Participants are also provided with a book on mindfulness accompanied by a guided meditation CD for personal use (see Ref. 11 for more intervention details). Mindfulness practice is assigned as homework beginning with 5 minutes daily then advances to 20 minutes daily by final session. The MAPs program has been shown in previous work to attenuate psychological stress and increase levels of mindfulness in a community-based setting.^14^

### Image Acquisition and Analysis

All brain images were acquired on a 1.5 T Siemens Sonata scanner (Erlangen, Germany) using an 8-channel head coil and a T1-weighted MPRAGE sequence with the following parameters: 1900 ms TR, 4.38 ms TE, 15° flip angle, 160 contiguous sagittal slices, 256x256 mm^2^ FOV, 1x1x1 mm^3^ voxel size. Brain images were processed using the SPM8 software (www.fil.ion.ucl.ac.uk/spm) and the VBM8 toolbox (dbm.neuro.uni-jena.de/vbm.html) with the protocol for longitudinal studies as previously described^15^ wherein images were smoothed with an 8 mm FWHM kernel. Voxel-wise gray matter was compared at baseline (t1) and follow-up (t2) using a paired t-test in SPM8. Results were corrected for multiple comparisons by controlling the Family-Wise Error (FWE) at cluster-level using a threshold of *p* ≤ 0.05, adjusted for non-stationarity.^16^

## RESULTS

**Figure 1:**
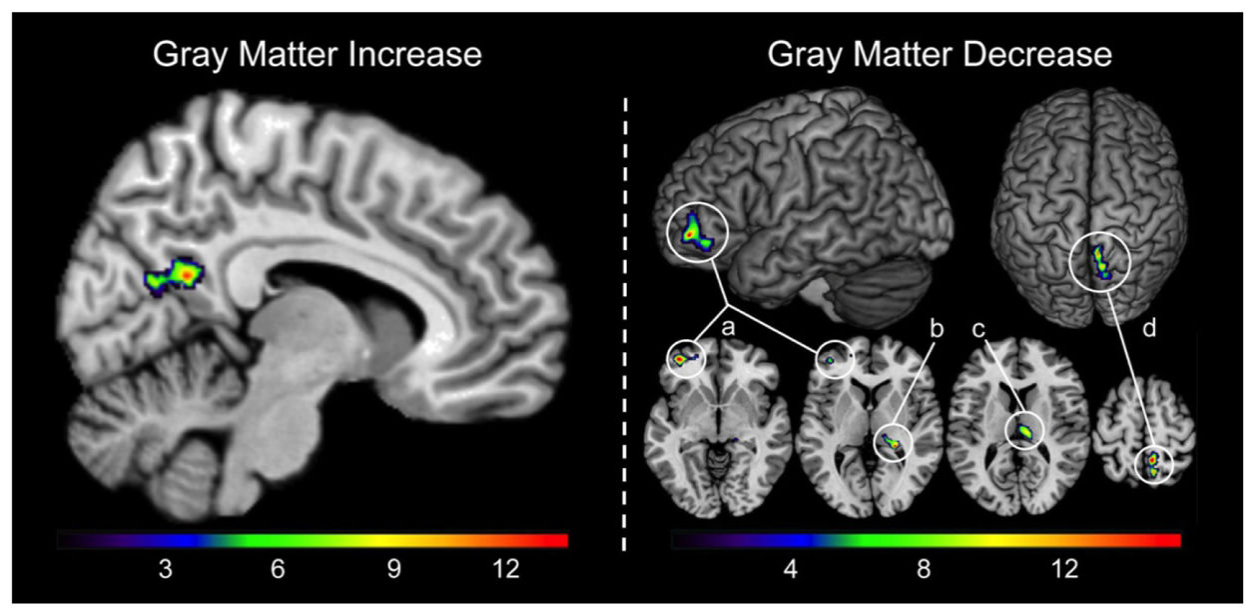
Significant increase and decrease in gray matter over the mindfulness meditation intervention period; threshold at p= 0.05 (corrected for multiple comparisons). The color bar encodes T-values. Significant gray matter increase was located in the right precuneus. Significant gray matter decreases were located in (a) the left prefrontal cortex, (b) the right hippocampus, (c) the right thalamus, and (d) the right parietal cortex.

As shown in Figure 1, the voxel-wise analysis revealed one cluster indicating significant gray matter increase in the right precuneus (247 voxels, cluster maximum [x;y;z]: 6; −64; 19, p=2.4x10^−15^) and decreases in the left prefrontal cortex (408 voxels, x;y;z: −39; 50; −8, p=1.0x10^−10^), right hippocampus (136 voxels, x;y;z: 24; −36;3, p=1.3 x 10^−7^), right thalamus (199 voxels, x;y;z:3;−21;12, p=1.7x10^−5^), and right parietal cortex (311 voxels, x;y;z: 9;−45;58, p=1.8x10^−15^).

## DISCUSSION

Outcomes from this exploratory, data-generating, study demonstrate that after the course of a six-week standardized mindfulness meditation program, significant changes in local gray matter were observed in older adults with sleep complaints. Although mindfulness-induced gray matter changes have been detected and described previously,^5,6^ the current findings are particularly interesting given the mature age of the participants. Gray matter changes due to MBIs were previously reported in a group of young and healthy, albeit stressed, individuals.^5,6^ Such changes in brain anatomy observed in young populations, however, cannot be easily extrapolated to older adults. Our observed gray matter increase within the precuneus in the older adult brain corroborates a previous longitudinal MBI study, in which the cluster peak voxel was located in the neighboring posterior cingulate cortex.^6^ The cluster identified in our study, as well as the one from the previous mindfulness meditation study, are located in the posterior part of the default mode network,^17^ which is implicated in meditation training.^18^ The precuneus is central to the human experience of the phenomenological self, a process proposed to be essential to meditation practice.^19^ Although unanticipated, gray matter decreases observed in this study have been reported previously^5^ and in our study may constitute effects that are specific to the older age of our participants and/or to the brain architecture subserving the remediation of sleep complaints. As such, findings might not generalize to asymptomatic or younger adults. This study highlights the need for future research to investigate neuroplastic changes that are associated with mindfulness meditation in older adults. This new line of investigation is promising when considering the recent experimental research showing that mind-body practices can reduce psychological ailments in older adults while also modulating immune cell parameters.^3,4^

Limitations of this exploratory study include the pre-post observational design and lack of a control group, which limits interpretation of causal inference. Sample size was small and all participants were Caucasian and reported current sleep disturbances, thus statistical noise is a potential explanation for the results and the generalizability of findings is curtailed. Future controlled studies enrolling adequate sample sizes are needed to replicate our preliminary results in older adults.
